# Molecular Epidemiology of Na^+^-Taurocholate Cotransporting Polypeptide Deficiency in Guangdong Province, China: A Pilot Study by Screening for Four Prevalent Variants of the Causative Gene *SLC10A1*

**DOI:** 10.3389/fgene.2022.874379

**Published:** 2022-04-27

**Authors:** Hua Li, Rong Chen, Gui-Zhi Lin, Wei-Xia Lin, Muhammad-Rauf Yaqub, Yuan-Zong Song

**Affiliations:** Department of Pediatrics, The First Affiliated Hospital, Jinan University, Guangzhou, China

**Keywords:** NTCP deficiency, epidemiology, SLC10A1 gene, Guangdong, prevalent variants

## Abstract

Na^+^-taurocholate cotransporting polypeptide deficiency (NTCPD) is an autosomal recessive disorder arising from biallelic *SLC10A1* mutations. As a newly-described inborn error of bile acid metabolism, the epidemiology of this condition remains largely unclear in Chinese population so far. In this study, a total of 2,828 peripheral blood samples were collected from 12 cities in Guangdong, a province with the largest population in China, and the four prevalent *SLC10A1* variants c.800C > T (p.Ser267Phe), c.263T > C (p.Ile88Thr), c.595A > C (p.Ser199Arg) and c.665T > C (p.Leu222Ser) were screened for by using polymerase chain reaction (PCR)- restriction fragment length polymorphism (RFLP). As a result, 663 mutated *SLC10A1* alleles were detected, and the mutated allele frequency was calculated to be 11.72% (663/5,656), with a carrier frequency 20.69% (1/5) and a theoretical morbidity rate 1.37% (1/73) of NTCPD in Guangdong province. The variant c.800C > T (p.Ser267Phe) exhibited highest allele frequency among the four prevalent variants (χ^2^ = 1501.27, *p* < 0.0001) as well as higher allele frequency in the peripheral region than that within the Pearl River Delta (χ^2^ = 4.834, *p* < 0.05). The results suggested that NTCPD might be a disorder rather common in Guangdong province. The findings depicted the molecular epidemiologic features of NTCPD, providing preliminary but significant laboratory evidences for the subsequent NTCPD diagnosis and management in Guangdong population.

## Introduction

Na^+^-Taurocholate Cotransporting polypeptide deficiency (NTCPD) is an autosomal recessive disorder affecting the hepatic uptake of bile acids caused by biallelic variants of the solute carrier family 10 member1 (*SLC10A1*) gene which encodes NTCP protein. The main clinical presentation of this disease is refractory hypercholanemia, transient cholestatic jaundice in infancy, and indirect hyperbilirubinemia in neonates (Deng et al., 2021; Deng, 2021). The gene *SLC10A1* is located at chromosome 14q24.2, contains five exons and has a total length of 23 kb (Hagenbuch and Dawson, 2004; Shiao et al., 2000). The protein product NTCP is expressed in the sinusoidal plasma membrane of the hepatocyte, where it functions to uptake bile salts from plasma in a Na^+^-dependent manner, playing a crucial role in the enterohepatic circulation of bile acids (Hagenbuch and Dawson, 2004; Anwer and Stieger, 2014).

Although *SLC10A1* gene was cloned by (Hagenbuch and Meier, 1994), the first case of NTCPD was just reported by (Vaz et al., 2015), and since then, increasing number of such patients have been reported (Yan et al., 2020). However, some patients were overinvestigated and intervented due to unclear etiology, even undergoing surgical operation such as exploratory laparotomy (Li et al., 2018; Deng, 2021), while pregnant women with NTCPD were sometimes misdiagnosed with intrahepatic cholestasis of pregnancy (ICP) and given cesarean section (Chen et al., 2019). Currently, the *SLC10A1* genotypic and phenotypic features of NTCPD still remain far from being well-understood.

Previous studies revealed that the allele frequency of c.800C > T (p.Ser267Phe), a pathogenic variant of *SLC10A1* gene (Deng et al., 2016; Ho et al., 2004), varied greatly among different ethnic groups and geographical populations. It was the most prevalent variant in South China and Vietnam, but was not found in European Americans, African Americans or Hispanics (Peng et al., 2015). Of note, in a sample of 50 Chinese Americans, the allele frequency of the variant was 7.5% (Ho et al., 2004), suggesting that NTCPD may not be rare in China. However, as a newly-described inborn error of metabolism, the epidemiology of NTCPD remains largely unclear in Chinese population.

From June 2015 to September 2021, our team diagnosed 318 NTCPD patients by *SLC10A1* gene analysis in China, and the four *SLC10A1* variants of c.800C > T (p.Ser267Phe), c.263T > C (p.Ile88Thr), c.595A > C (p.Ser199Arg) and c.665T > C (p.Leu222Ser) were at the top of the list, accounting for 96.3, 2.5, 0.6 and 0.4% of all mutated alleles, respectively (Deng, 2021; Deng et al., 2021). In this study, these *SLC10A1* variants were screened to investigate the epidemiology of NTCPD in Guangdong, a province with the largest population in China.

## Methods

### Participants

According to the latest data from the seventh national Census of China’s National Bureau of Statistics, the permanent resident population in Guangdong were 126,012,510 (http://www.stats.gov.cn/tjsj/tjgb/rkpcgb/qgrkpcgb/202106/t20210628_1818822.html). In order to achieve statistical significance, the sample size was calculated to be at least 1,537 on the basis of the estimated carrier rates of 1/50 by using the online sample size calculator EPITOOLS (https://epitools.ausvet.com.au/oneproportion), with a confidence level of 95% and the precision of 0.02. The number of samples from each region was in accordance with the population distribution.

**FIGURE 1:**
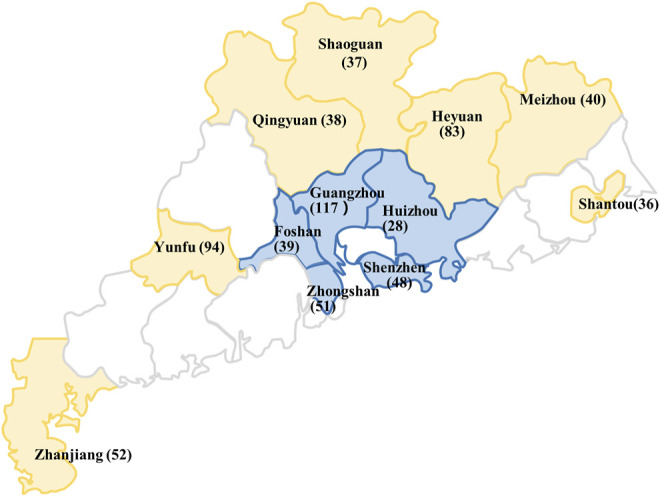
Regional division and the distribution of the mutated *SLC10A1* alleles in different cities of Guangdong province, including the cities within the Pearl River Delta (blue) and the peripheral region (yellow). The numbers of the mutated *SLC10A1* alleles from different cities were presented in parentheses. This figure was generated by means of the software Microsoft PowerPoint 2019. The base map was created by incrementally assembling the outlines of the Chinese administrative regions, which could be downloaded *via* the URL link http://www.900ppt.com/.

Inclusion criteria: Apparently healthy individuals regardless of their genders and ages. The research subjects in this study were 2,828 used blood samples for health examination collected from 12 different cities in Guangdong Province from April 2011 to March 2013. Among them, there were 476, 276, 189, 169 and 192 samples collected from the five cities Guangzhou, Shenzhen, Foshan, Huizhou and Zhongshan, respectively, within Pearl River Delta region. The remaining samples were from the seven peripheral cities to the Pearl River Delta, with 162, 328, 178, 147, 181, 333, and 197 samples collected from Meizhou, Heyuan, Zhanjiang, Qingyuan, Shaoguan, Yunfu, and Shantou, respectively (Figure 1).

Exclusion criteria: Patients with any positive symptoms or signs including jaundice or hepatosplenomegaly.

### Genetic Analysis

**FIGURE 2:**
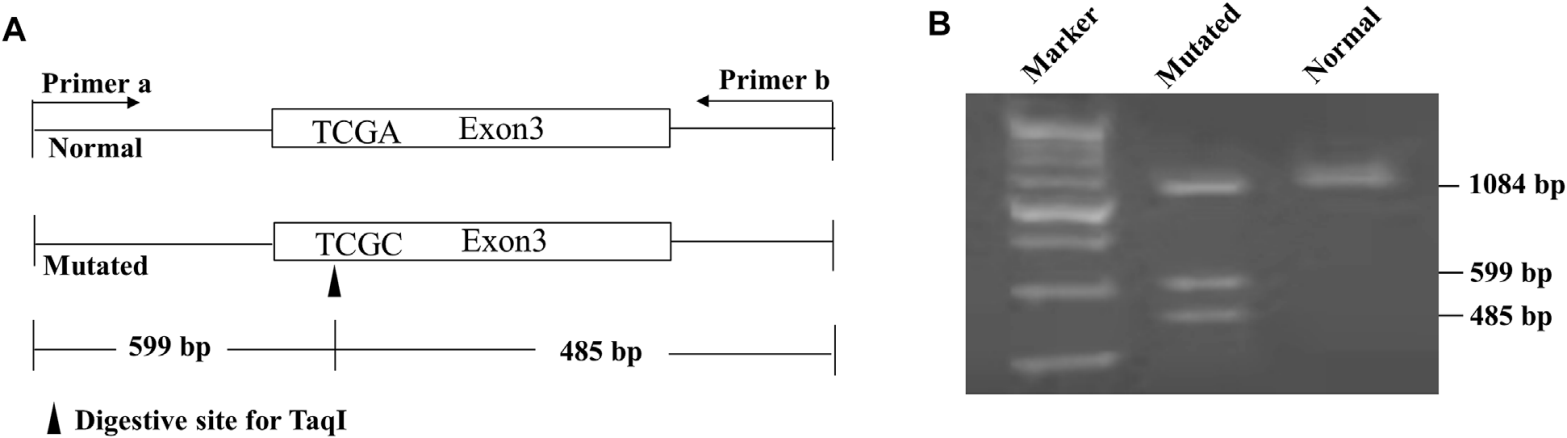
Novel approach developed for the screening of the *SLC10A1* variant c.665T > C (p. Leu222Ser). **(A)** schematic diagram of the PCR-RFLP approach. The mutated *SLC10A1* allele had a TaqI restriction enzyme site and produced the 599 and 485 bp fragments from the 1,084 bp band following enzymatic digestion. **(B)** Gel electrophoresis showed that the heterozygote of the variant c.665T > C (p. Leu222Ser) had three bands of 1084, 599, and 485 bp, while the wildtype had only one band of 1084 bp.

As in previous publications, genomic DNA was purified from the peripheral blood samples, and the variants c.800C > T (p.Ser267Phe) (Deng et al., 2016), c.263T > C (p.Ile88Thr) (Qiu et al., 2017) and c.595A > C (p.Ser199Arg) (Li et al., 2019) were detected using established polymerase chain reaction (PCR)-restriction fragment length polymorphism (RFLP) methods. The variants c.665T > C (p. Leu222Ser) was screened using a novel PCR-RFLP procedure as below. The nucleotide sequences of the forward and reverse primers in PCR amplification were 5′-GTG​CTT​GGC​TGA​GTT​TGT​AAT​AAT​C-3′ and 5′- GTG​TTT​GGA​TAC​CTT​TGG​TGT​CTG-3′, respectively (Invitrogen; Thermo Fisher Scientific, Inc.). The target fragment was amplified using a PCR kit (Takara Biotechnology Co., Ltd.) and the PCR thermocycling conditions were: 94°C for 5 min, followed by 35 cycles at 94°C for 30 s, 58°C for 40 s and 72°C for 50 s, and 72°C for 10 min. The TaqI restriction enzyme (Thermo Fisher Scientific, inc.) was used to digest the PCR products and the digested DNA products were subsequently separated by electrophoresis in a 4% agarose gel (Figure 2).

### Calculation of the Allele Frequencies, Carrier Frequencies and NTCPD Morbidity Rate

The mutated *SLC10A1* allele frequencies, carrier frequencies and NTCPD morbidity rate in different areas were calculated based on the Hardy-Weinberg equilibrium. The genotypes *AA* (healthy individual), *AB* (carrier) and *BB* (patient) of a biallelic genetic marker were expected to have the relative frequencies of *p* ^*2*^, *2pq* and *q* ^*2*^, with p and q being the *A* (wild type) and *B* (mutant) allele frequency, respectively; and thus *p* + *q* = 1. The values of 2*pq* and *q* ^*2*^ represented as the carrier frequency of *SLC10A1* variants and NTCPD morbidity rate, respectively (Graffelman et al., 2017; Graffelman and Weir, 2018).

### Statistical Analysis

By using the statistical software SPSS version 26.0 (IBM Corp., Chicago, IL, United States), the distribution of the four *SLC10A1* variants was compared *via* Chi-square tests among different geographic regions, with a *p* value < 0.05 indicating statistical significance.

This study was conducted in accordance with the Declaration of Helsinki (as revised in 2013) and was approved by the Medical Ethics Committee of the First Affiliated Hospital, Jinan University, Guangdong, China (No.KY-2019-052). Participants were genotyped retrospectively using blood samples previously collected for the purpose of health examination. The data related to individual identification were anonymized during the entire study process. Therefore, informed consent was waived.

## Results

### Variants Screening

This study detected 544 heterozygous and 20 homozygous variants of c.800C > T (p.Ser267Phe), 62 heterozygotes of c.263T > C (p. Ile88Thr), four heterozygotes of c.595A > C (p. Ser199Arg) and three heterozygotes of c.665T > C (p. Leu222Ser), along with five compound heterozygote of c.800C > T (p. Ser267Phe) and c.263T > C (p. Ile88Thr). Therefore, a total of 663 mutated *SLC10A1* alleles were detected in 5,656 independent alleles (2,828 samples), as shown in Table 1.

**TABLE 1:** Distribution of the four prevalent SLC10A1 variants in different regions of Guangdong Province.

Regions	Cities	Number of samples	*SLC10A1* Variants					Mutated alleles	Allele frequencies (%)	Carrier frequencies (%)	Morbidity rates (%)
			c.800C > T heterozygote/homozygote	c.263T > C	c.800C > T/c.263T > C	c.595A > C	c.665T > C				
Pearl River Delta	Guangzhou	476	85/6	12	4	0	0	117	12.29	21.56	1.51
	Shenzhen	276	41/0	5	0	0	2	48	8.70	15.89	0.76
	Zhongshan	192	37/3	8	0	0	0	51	13.28	23.03	1.76
	Huizhou	169	27/0	1	0	0	0	28	8.82	15.19	0.69
	Foshan	189	34/0	5	0	0	0	39	10.32	18.51	1.07
	Total	1,302	224/9	31	4	0	2	283	10.87	19.38	1.18
Periphery of the PearlRiver Delta region	Meizhou	162	38/0	2	0	0	0	40	12.35	21.65	1.53
	Heyuan	328	72/1	9	0	0	0	83	12.65	22.10	1.60
	Zhanjiang	178	41/2	5	1	0	0	52	14.61	24.95	2.13
	Qingyuan	147	32/2	2	0	0	0	38	12.93	22.52	1.67
	Shaoguan	181	29/3	2	0	0	0	37	10.22	18.35	1.04
	Yunfu	333	76/3	7	0	4	1	94	14.11	24.24	1.99
	Shantou	197	32/0	4	0	0	0	36	9.14	16.61	0.84
	Total	1,526	320/11	31	1	4	1	380	12.45	21.80	1.55
In total		2,828	544/20	62	5	4	3	663	11.72	20.69	1.37

### Carrier Frequencies and Theoretical Morbidity Rates

The mutated *SLC10A1* allele frequency in the Guangdong was calculated to be 11.72% (663/5,656), the carrier frequency, 20.69% (1/5), and the theoretical morbidity rate 1.37% (1/73), respectively. According to China’s seventh national census population data and the theoretical morbidity rate, it was estimated that there were at least 1,726,371 NTCPD patients in Guangdong province.

The mutated *SLC10A1* allele frequency in the Pearl River Delta was 10.87% (283/2,604), with a carrier frequency 19.38% (1/5), and a theoretical morbidity 1.18% (1/85); in the peripheral region to Pearl River Delta, the mutated *SLC10A1* allele frequency was 12.45% (380/3,052), with a carrier frequency 21.80% (1/5) and a theoretical morbidity rate 1.55% (1/65) (Table 1).

### Distribution Comparison of the Prevalent SLC10A1 Variants Among Different Geographic Regions

The allele frequency of c.800C > T (p.Ser267Phe) was the highest among the four prevalent variants (Table 2). The allele frequency of the variant c.263T > C (p. Ile88Thr), c.595A > C (p. Ser199Arg) and c.665T > C (p. Leu222Ser) between the Pearl River Delta region and the periphery of the Pearl River Delta had no significant difference; However, the distribution of the variant c.800C > T (p. Ser267Phe) was significantly different between the two regions, and the variant frequency (9.45%, 246/2,604) in the periphery of the Pearl River Delta region was higher than that (11.24%, 343/3,052) in the Pearl River Delta region (χ^2^ = 4.834, *p* < 0.05) (Table 3).

**TABLE 2:** The allele frequency of the four prevalent *SLC10A1* variants in Guangdong population.

Prevalent variants	*SLC10A1* alleles		Mutated allele frequencies	Chi squares	*p* values
	Mutated	Wild-type			
c.800C > T (p. Ser267Phe)	589	5,067	10.41%(589/5,656)		
c.263T > C (p. Ile88Thr)	67	5,589	1.18% (67/5,656)	440.94^a^	<0.0001^a^
c.595A > C (p. Ser199Arg)	4	5,652	0.07% (4/5,656)	609.03^a^	<0.0001^a^
c.665T > C (p. Leu222Ser)	3	5,653	0.05% (3/5,656)	612.09^a^	<0.0001^a^
Total	663	4,993	11.72% (663/5,656)	1501.27	<0.0001

a Compared with the group c.800C > T, respectively.

**TABLE 3:** Comparison of the distribution of the four prevalent *SLC10A1* variants between the Pearl River Delta and the peripheral region.

Regions	*SLC10A1* Variants							
	c.800C > T (p.Ser267Phe)		c.263T > C (p.Ile88Thr)		c.595A > C (p.Ser199Arg)		c.665T > C (p.Leu222Ser)	
	Mutated	Wild-type	Mutated	Wild-type	Mutated	Wild-type	Mutated	Wild-type
Pearl River Delta	246	2,358	35	2,569	0	2,604	2	2,602
Peripheral region	343	2,709	32	3,020	4	3,048	1	3,051
Chi squares	4.83		1.049		3.41		0.51
*p* values	0.028		0.306		0.06		0.47

## Discussion

For the first time, this study reported that the allele frequency of four prevalent *SLC10A1* variants in Guangdong was 11.72% (663/5,656), with a carrier frequency 20.69% (1/5) and a theoretical morbidity rate 1.37% (1/73) of NTCPD in this province. According to the latest data from the seventh national population census in 2020, the population in Guangdong reached 126,012,510 (http://www.stats.gov.cn/tjsj/tjgb/rkpcgb/qgrkpcgb/202106/t20210628_1818822. html), and thus it was estimated that at least 1,726,371 NTCPD patients were distributed in Guangdong province. Although no relevant data have been reported in terms of the actual incidence or diagnosis rate of NTCPD, the findings in this study provided preliminary but significant data for the estimation of the NTCPD burden, constituting an important epidemiologic basis for the diagnosis and management of NTCPD in Guangdong, and so high a theoretical morbidity implied that a large number of NTCPD patients might have been overlooked or even misdiagnosed at least in Guangdong population.

Among the four prevalent variants in this study, c.800C > T (p.Ser267Phe) had the highest allele frequency, accounting for 89% (589/663) of the total variants. NTCP was also a functional receptor for human hepatitis B virus (HBV) (Yan et al., 2012), and this variant not only affected the NTCP residue critical for bile salts binding, but also severely impaired viral infection by HBV (Yan et al., 2014). Actually, HBV infection was rather common in Guangdong population. In 1992, the positive rate of HBsAg was as high as 17.85% in this province, while the national average was just 9.75% (Wu et al., 2009). Even in 2006, the positive rate of HBsAg reached 17.55% in Guangdong population aged 15 ∼ 59 years (Cui et al., 2012). Therefore, the high allele frequency of c.800C > T (p. Ser267Phe) in Guangdong might be a result of positive selection in response to HBV infection (Peng et al., 2015). Actually, this variant was associated with a lower incidence of acute-on-chronic liver failure (ACLF) in chronic hepatitis B (CHB) patients (Peng et al., 2015) and reduced the risk for HBV infection and disease progression in human (An et al., 2018). Moreover, the c.800C > T (p.Ser267Phe) variant exhibited relatively higher allele frequency (Table 2), which could be explained by a founder effect of this variant which had originated in a far remote ancestor in the long process of evolution.

This study showed that the allele frequency of c.800C > T (p.Ser267Phe) was higher in the periphery region than that within the Pearl River Delta region. Guangdong is one of the main inflow places of China’s migrant population. In 2010, the migrant population reached 31.28 million, mainly from Hunan, Guangxi, Sichuan, Hubei and other provinces, and nearly 90% of the migrant was distributed in Guangzhou, Shenzhen, Dongguan, Foshan and other cities in the Pearl River Delta region (Chen, 2017). Since the HBsAg carrier rate in these migrant source provinces was lower than that in Guangdong province (Cui et al., 2012; Duan, 2018), it was not surprising that the *SLC10A1* variant frequency in the Pearl River Delta region was lower than that in the periphery of the Pearl River Delta region dominated by local residents. In other words, the different geographic distribution of the variant c.800C > T (p. Ser267Phe) might be attributed to the genetic flow which occurred between distinct founding populations.

Interestingly, 20 homozygotes of c.800C > T (p.Ser267Phe) and five compound heterozygotes of this variant with c.263T > C (p. Ile88Thr) were found in this study, who had no clinical signs and symptoms such as jaundice and hepatosplenomegaly. This is not strange since similar findings have been reported that, in affected adults, NTCPD only presented with laboratory abnormalities including hypercholanemia while the clinical presentation was usually negative (Deng et al., 2016; Liu et al., 2017), although this disease caused abnormal bilirubin metabolism in pediatric patients (Yan et al., 2020), and increased the risks of indirect hyperbilirubinemia in affected neonates as well as transient cholestatic jaundice, elevated liver enzymes and 25-hydroxyvitamin D (Vit D) deficiency during early infancy (Deng et al., 2021).

There were still some limitations in this paper. This study only focused on four prevalent *SLC10A1* variants in populations from 11 cities in Guangdong Province, and sampling bias might exist with limited sample sizes in relevant cities. Therefore, further epidemiology study focusing on more *SLC10A1* variants with larger sample size was in need.

## Conclusion

NTCPD, with a theoretical morbidity rate of 1.37%, might be a disorder rather common in Guangdong province, and the prevalent *SLC10A1* variant c.800C > T (p. Ser267Phe) exhibited significantly different distribution between Pearl River Delta and the peripheral regions. The findings depicted the molecular epidemiologic features of NTCPD in Guangdong population, providing preliminary but significant laboratory evidences for subsequent NTCPD diagnosis and management in this province.

## Data Availability

The original contributions presented in the study are included in the article/Supplementary Material, further inquiries can be directed to the corresponding author.
